# Development of a gestational diabetes risk assessment scale: a validity and reliability study

**DOI:** 10.55730/1300-0144.5755

**Published:** 2023-10-12

**Authors:** Selma DAĞCI, Besey ÖREN

**Affiliations:** 1 İstanbul Provincial Health Directorate, İstanbul, Turkiye; 2 Department of Internal Medicine Nursing, Faculty of Health Sciences, University of Health Sciences, İstanbul, Turkiye

**Keywords:** Gestational diabetes mellitus, risk assessment, scale development, validity and reliability

## Abstract

**Background/aim:**

The aim of this study was to develop a gestational diabetes risk assessment scale (GEDRISK).

**Materials and methods:**

This methodological study included 652 pregnant women who presented to six public health institutions in İstanbul between September 2021 and February 2022. Content validity was evaluated using the content validity index, while construct validity was assessed through exploratory and confirmatory factor analyses. Item discrimination was examined using Cronbach’s alpha coefficients, Spearman–Brown and Guttman coefficients, item–total correlation tests, and 27% lower and upper quartile t tests. Reliability was determined through test–retest analysis methods.

**Results:**

The scale-level content validity index demonstrated strong coherence at 0.83, confirming its robustness. In the EFA, the scale, comprising 18 items and 5 subdimensions, accounted for 57.48% of the variance (n = 652). The results of the confirmatory factor analysis were as follows: χ^2^/df = 2.28; RMR = 0.01; CFI, GFI, and IFI = 0.95; AGFI = 0.93; NFI = 0.92; RFI = 0.90; and RMSEA = 0.04. The Spearman–Brown and Guttman split-half coefficient equal length analysis produced a result of 0.826, Cronbach’s alpha value was 0.756, and the temporal consistency of the scale was evaluated with the test–retest method (p = 0.184). The structure of the scale was evaluated with a validity and reliability analysis and was found to have acceptable, valid, and reliable properties. The mean total GEDRISK score of the pregnant women participating in the study was 33.57 ± 4.71. It was observed that the GEDRISK scale identified 51% of the respondents diagnosed with diabetes through an oral glucose tolerance test.

**Conclusion:**

The GEDRISK scale was found to be a valid and reliable tool for the measurement of gestational diabetes risk in a sample of the Turkish population.

## 1. Introduction

Gestational diabetes mellitus (GDM) is an endocrine disease defined as carbohydrate intolerance in the absence of type I or type II diabetes mellitus (DM). It occurs in the second or third trimester of pregnancy and disappears after pregnancy [1–3]. According to the 2019 data published by the International Diabetes Federation, the prevalence of hyperglycemia is thought to increase with age, and pregnancy-induced hyperglycemia is particularly prominent in low- and middle-income countries where GDM is diagnosed in 50%–70% of cases [4].

Gestational diabetes mellitus is a significant public health problem that is often overlooked, yet it has a profound impact on both maternal and child health. It can lead to an increased risk of gestational hypertension, preeclampsia, macrosomia, and the need for Cesarean section among the women, as well as an increased likelihood of type II DM and obesity in the babies later in life [4,7]. An early diagnosis of GDM can aid in preventing the fetal and maternal complications mentioned in the literature, while also promoting overall health improvement.

To identify pregnant women at high risk of GDM, the American Diabetes Association recommends the use of fasting plasma glucose, HbA1c, and oral glucose tolerance tests (OGTT) [1]. However, in addition to necessitating intervention, these tests are time-consuming and can incur significant costs when applied to large populations.

Numerous country-specific risk assessment methods have been described in the literature for measuring the risk of type II DM on a national basis, such as the Australian, Danish, Indian, and German diabetes risk scores, among others [8–14]. However, a literature review highlights the absence of a standard approach for identifying GDM risk without necessitating intervention.

Pregnant women at high risk of GDM are currently identified only through OGTT screening studies. Consequently, there is a need for a method similar to the type II DM risk score in Turkey that would enable primary healthcare institutions or individuals to assess their own GDM risk without requiring intervention. The gestational diabetes risk assessment (GEDRISK) scale allows pregnant women who opt not to undergo the OGTT, encounter difficulties in getting tested, or face challenges in visiting a health facility to be evaluated to become aware of the risks of GDM. The present study, which can serve as a guide for pregnant women and healthcare professionals in the GDM risk assessment process, aims to develop an easily applicable and cost-effective tool that is both valid and reliable for determining GDM risk within Turkish society.

## 2. Materials and methods

This methodological study introduces a valid and reliable measurement tool for assessing the risk of GDM in pregnant women.

### 2.1. Sample size estimation

The study population consisted of 5 training and research hospitals and a state hospital operating under the Ministry of Health in the Anatolian Region of İstanbul. All of the medical facilities have diabetes outpatient clinics and active delivery rooms, and they serve a large number of pregnant women for maternal procedures. The study was conducted between September 2021 and February 2022 and focused on a sample of pregnant women between 24 and 28 weeks of gestation. These women were required to be proficient in Turkish, be aged 18 years or older, have undergone OGTT, and have consented to participate in in the study.

For validity and reliability studies, it is recommended to have a sample size that is 5–10 times larger than the number of items in the scale [15–18]. Accordingly, a sample size of 50 participants is considered very insufficient, a sample of 100 participants is deemed insufficient, a sample of 200 participants is regarded as reasonable, a sample of 300 participants is considered good, a sample of 500 participants is considered very good, and a sample of 1000 participants is considered excellent [18–20]. The sample in the present study included 652 pregnant women between 24 and 28 weeks of gestation who met the study inclusion criteria and who consented to participate in the study. The test–retest analysis carried out within the scope of the reliability studies involved 41 pregnant women who were not included in the study sample at the factor analysis stage.

### 2.2. Data collection

A pregnancy information form, the draft GEDRISK scale, and the OGTT were used for the collection of data for the study. All of the data collection tools were filled out during face-to-face interviews with the participants. The pregnancy information form consists of 4 sections designed to collect data on various factors: (a) unalterable risk factors, including genetics [21], ethnicity [22], age [23], height [24], family history of DM [25], presence of polycystic ovary syndrome [23], history of fetal macrosomia [26], and stillbirth; (b) alterable risk factors, such as impaired glucose tolerance, history of GDM, fasting blood glucose levels >95 mg/dL, HbA1c levels higher than 5.7%, excessive weight gain in early pregnancy (2 kg for the first trimester, 0.6 kg/week for the second trimester for low-weight pregnant women, 0.45 kg/week for normal weight pregnant women, and 0.27 kg/week for obese pregnant women), weighing 10% or more greater than normal body weight, and a body mass index exceeding 30 kg/m^2^; and (c) modifiable risk factors, such as smoking, history of hypertension, multiparity, an unhealthy diet (rich in red meat and processed meat products), and lack of physical activity [23,27–32].

#### Preliminary GEDRISK scale

This scale was developed for pregnant women to self-assess their risk of GDM. The studies used to create the item pool were searched in PubMed, the Council of Higher Education National Dissertation Center, Scopus, Ulakbim, Web of Science, and Science Direct databases using the keywords “gestational diabetes mellitus”, “diabetes”, “GDM risk factors”, “GDM modifiable risk factors”, and “GDM non-modifiable risk factors”. The preliminary scale was designed as a 3-point Likert-type scale, offering respondents the options of “yes”, “sometimes/I don’t know”, and “no”. It consists of 53 items, including both negative and positive statements.

### 2.3. Statistical analysis

The data obtained in the research were evaluated in a computer environment using the IBM SPSS Statistics for Windows (Version 25.0. Armonk, NY: IBM Corp.) and Amos 24 statistical programs. Surface, content, construct validity, and reliability analyses were used to assess the validity and reliability of the GEDRISK scale.

#### 2.3.1. Validity

Validity refers to the extent to which a measurement tool under development can accurately measure the characteristics it intends to assess within the target group, without causing confusion with other characteristics [33]. Expert opinion was sought for the content validity of the scale, and a factor analysis was carried out for the assessment of construct validity. The content validity assessment of the preliminary scale consisted of 4 phases: expert selection, preparation of a content validity expert evaluation form, collection of expert opinions, and determination of the scale items. The preliminary GEDRISK scale was presented for review to 70 experts determined by the researchers as having carried out studies on DM and GDM. Subsequently, the researchers received input from 36 experts, including professors, associate professors, assistant professors, and specialist doctors employed in departments of internal medicine, obstetrics/gynecology, or endocrinology. The experts evaluated the scale items using 3 response options: the item measures the targeted structure (3 points), the item is related to the structure but is unnecessary (2 points), and the item does not measure the targeted structure (1 point). Subsequently, using the method developed by Lawshe (1975), the content validity ratio (CVR) for each of the scale items and the content validity index (CVI) for the entire scale was calculated based on the scores given by the experts [34].

A principal component analysis and the varimax method were used for the exploratory factor analysis (EFA) in the present study. Prior to conducting the EFA, Kaiser–Meyer–Olkin (KMO), Bartlett’s, and antiimage correlation tests were carried out as preliminary assumption tests for the applicability of the analysis.

A confirmatory factor analysis (CFA) was performed to determine the compatibility of the factors obtained after the EFA with the real structure. The CFA is a hypothesis-based analysis that allows for the examination of many analytical possibilities not covered in the EFA stage [35–37]. In scale development studies, the purpose of model building in CFA is to decide which subdimension the scale items will be placed into [38].

#### 2.3.2. Reliability

In Likert-type scales, the alpha coefficient is frequently calculated to assess the reliability of the scale from the total score to explain and question the homogeneous structure (internal consistency) of the items in the scale. In scale reliability studies, the assessment of internal consistency involves calculating Cronbach’s alpha coefficient, conducting a split-half analysis, and calculating an item–total score correlation coefficient. To assess the item discrimination of the scale, various methods were employed, including the lower and upper quarter t test at 27%, an analysis of variance Tukey’s nonadditivity analysis to examine the relationship and summability of the scale items, and a test–retest analysis to evaluate measurement invariance across time.

### 2.4. Ethical considerations

Written permission was obtained from the İstanbul Provincial Health Directorate to conduct the research in the designated health facilities. Additionally, approval was granted by the Scientific Research Ethics Committee of the University of Health Sciences through a letter dated 15.01.2021 and numbered 3086. The study was carried out in strict adherence to the principles outlined in the World Medical Association Declaration of Helsinki. Pregnant women who visited the hospitals involved in the study were, prior to any data collection, requested to complete a voluntary consent form signifying their willingness to participate in the research. Subsequently, the data were collected through face-to-face interviews. The respondents were assured that any information they shared with the researcher would be kept confidential, and utmost care was taken to protect their confidentiality.

## 3. Results

The mean age of the total sample was 29.1 ± 5.3 years, and a clear pluralityof the respondents had a high school level of education (36.0%). Further demographic characteristics of the respondents are presented in Table 1.

### 3.1. Validity

The CVR was calculated by analyzing the responses of the 36 experts for each item, using the CVR values detailed in the study conducted by Ayre and Scally (2014). This calculation yielded a value of 0.333 [39]. In the subsequent phase, 14 items with a CVR of 0 or below (negative) or less than 0.33 at the α = 0.05 significance level were removed from the scale. Based on the results of the content validity study, a preliminary scale of 39 items was constructed with a total CVI of 0.830.

Existing studies in the literature suggest conducting the test on a small sample that closely resembles the target group for which the scale was developed. Any refinements or analyses of the preliminary scale statements following this test are advised to involve either 5% of the sample or 30 individuals. Previous studies have been conducted with pilot tests involving 15–20 participants, but there is a lack of consensus regarding an acceptable sample size in this regard. In the present study, a pilot test was carried out with the involvement of 15 pregnant women who visited training and research hospitals on the Anatolian side of İstanbul. Feedback was sought from these participants to assess the clarity of the statements in the scale, the suitability of the scale for the intended purpose, the response method, and the quality of the statements. The preliminary scale underwent a surface validity assessment by the pregnant women, leading to the conclusion that modifications should be made to enhance the intelligibility of 4 items. Thus, the surface validity of the preliminary GEDRISK scale was established.

#### 3.1.1. Construct validity/factor analysis

The construct validity of the measurement tool was tested through exploratory and confirmatory factor analyses of the preliminary scale. In the present study, principal component analysis and the varimax method were used for the EFA, prior to the preliminary assumption tests, including the KMO, Bartlett’s, and antiimage correlation tests, to assess the applicability of the analysis. The preliminary GEDRISK scale demonstrated a KMO value of 0.77 (>0.60). Bartlett’s test yielded a result of χ^2^ = 3711.034, with a significance level of p < 0.001. The antiimage correlation values were within the range of 0.67–0.93. The Bartlett test of sphericity was used to test the hypothesis of whether the correlation matrix is a similar matrix and was rejected with a significance level of p < 0.001. In some instances, the first-factor solution resulting from the EFA may lack a simple and readable structure. In such cases, factors can be rotated into more interpretable positions through a process known as factor rotation. The evaluation of factor loading takes sample size into consideration, and the existing literature provides guidance on the number of acceptable factor loadings based on the size of the sample [35]. The sample size in the present study was 652, with an acceptable factor loading of 0.50.

Regarding construct validity, four varimax rotations were conducted during the principal component analysis of the EFA. Following the first rotation, 7 items were removed from the scale. In the second rotation, 4 items were removed, followed by 6 items in the third rotation and 4 items in the fourth rotation. Consequently, a total of 21 items with factor loadings of 0.50 and below were eliminated from the scale. There were no overlapping items in the scale, and as a result, the factor loading values for each item in the scale ranged from 0.559 to 0.926. The final version of the scale was formulated with 5 subdimensions and 18 items (Table 2).

After the EFA, all of the items were found to align perfectly with the subheadings/dimensions specified in Table 2. It was subsequently observed that, in the scree plot, the scale exhibited a noticeable decrease after the third factor, indicating a discontinuation in the structure of the scale.

**Figure 1:**
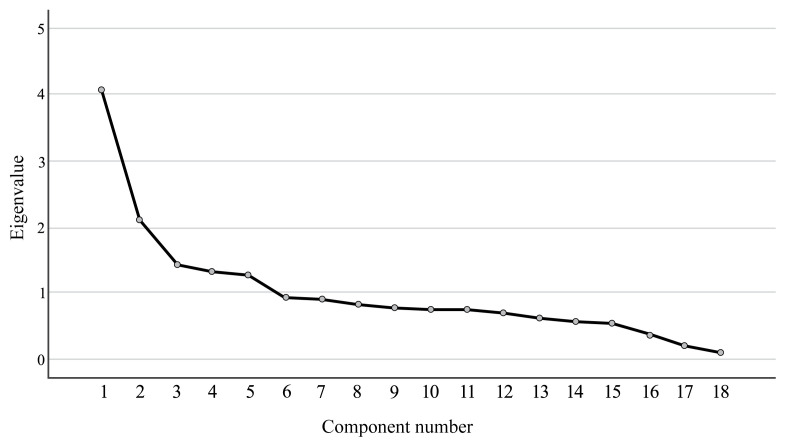
Gestational diabetes risk assessment scale scree plot.

This cutoff point validates the presence of 5 factors with eigenvalues exceeding 1 (Figure 1). These 5 subdimensions collectively accounted for 57.48% of the total variance of the scale; the first subdimension contributed 13.89%, the second 11.74%, the third 11.68%, the fourth 10.59%, and the fifth 9.56% to the explained variance (Table 2). In the subsequent phase, the initial factor labels that were established prior to the EFA underwent reevaluation. This assessment was based on a comprehensive review of the literature regarding GDM and its associated risk factors. Consequently, the decision was made to label the 5 subdimensions of the preliminary GEDRISK scale as infertility, insulin resistance before pregnancy, GDM-related measurements, pregnancy insulin resistance, and healthy lifestyle behaviors. It was observed that the subdimensions of the scale exhibited eigenvalues ranging from 1.16 to 4.20.

#### 3.1.2. Confirmatory factor analysis (CFA)

**Figure 2:**
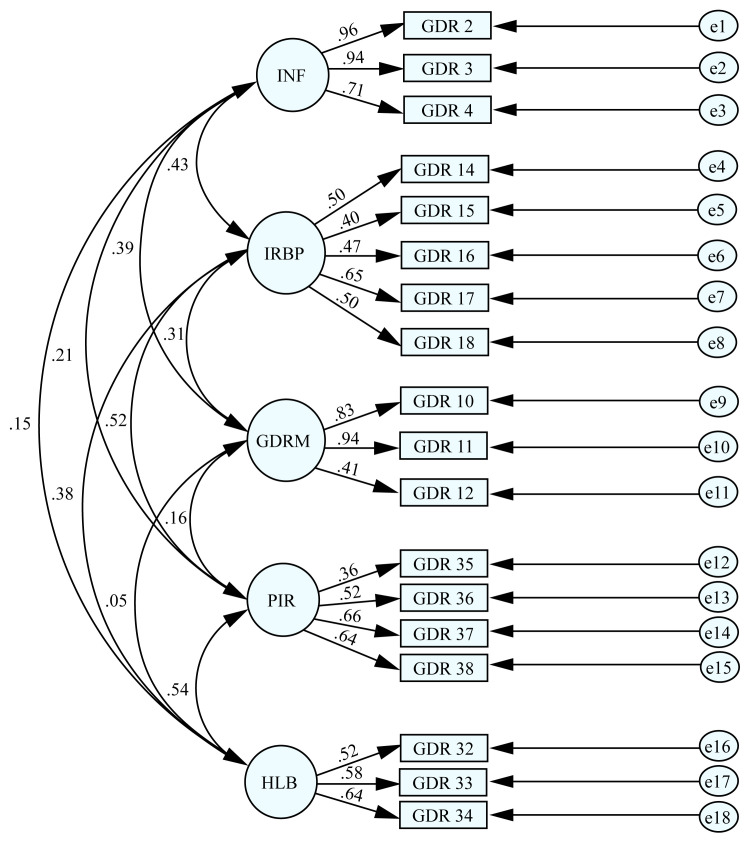
Path diagram for confirmatory factor analysis of the gestational diabetes risk assessment scale. INF = infertility, IRBP = insulin resistance before pregnancy, GDRM = GDM-related measurements, PIR = pregnancy insulin resistance, and HLB = healthy lifestyle behaviors.

The CFA carried out in this study yielded fit index values for the 5-factor structure that emerged following the EFA. Various fit indices were employed to validate the factor structure of the 18 item GEDRISK scale, including the adjusted chi-square statistic (χ^2^/df), goodness of fit index (GFI), comparative fit index (CFI), relative fit index (RFI), normed fit index (NFI), incremental fit index (IFI), adjusted goodness of fit index (AGFI), root mean square error of approximation (RMSEA), and root mean square residual (RMR) (Figure 2). The results of the CFA yielded the following: χ^2^/df = 2.28, RMSEA = 0.04, CFI = 0.95, RFI = 0.90, GFI = 0.95, NFI = 0.92, IFI = 0.95, RMR = 0.01, and AGFI = 0.93 (Table 3).

### 3.2. Reliability

#### 3.2.1. Internal consistency

In the second phase of the study, Cronbach’s alpha value for the scale was 0.756. After item removal, Cronbach’s alpha ranged between 0.739 and 0.756 (Table 4).

#### 3.2.2. 27% lower and upper quartile t test

As part of the item discrimination analysis, a t test was carried out on the lower 27% and upper 27% quartile groups of the GEDRISK scale scores. The results revealed a statistically significant difference between the average total scores of the GEDRISK scale for the upper and lower 27% quartiles (t = −32.65; p = 0.000). In terms of each individual subdimension, the t value was −9.30 for infertility, −37.59 for GDM-related measurements, −37.50 for insulin resistance before pregnancy, −57.22 for pregnancy insulin resistance, and −58.65 for healthy lifestyle behaviors. All t values were statistically significant with p = 0.000 in the respective subdimensions.

#### 3.2.3. Split-half analysis: Spearman–Brown and Guttman split-half test

In terms of scale reliability, an analysis of the correlation between the forms yielded a coefficient of 0.704, indicating a high level of reliability. Additionally, the Spearman–Brown and Guttman split-half coefficients were evaluated as reliability criteria, further confirming the high reliability of the scale (0.826).

#### 3.2.4. Analysis of variance Tukey’s nonadditivity test

The result of Tukey’s summability test indicated that the items comprising the scale were interconnected and homogeneous (F = 108.438, p < 0.001). This led to identifying the scale as an additive scale in terms of scoring (F = 5.815, p > 0.05).

#### 3.2.5. Item–total score reliability

The item–total correlation values, which assess the internal consistency of the scale and its item discrimination properties, ranged from 0.34 to 0.54 for the sample data. These values were found to be statistically significant (p < 0.05) (Table 4).

#### 3.2.6. Test–retest (invariance) reliability

To determine the invariance of the preliminary GEDRISK scale over time, the scale was administered to 41 pregnant women on 2 occasions, at a 2-week interval, using a test–retest method. The results showed a positive and statistically significant correlation between the scores obtained from the two measurements (r = 0.74, p < 0.001). Additionally, when the test–retest scores of the GEDRISK scale were compared, no significant difference was noted between the two applications (t = 1.352, p = 0.184, p > 0.05).

##### The final structure and evaluation of the GEDRISK scale

Following the validity and reliability analysis, the GEDRISK scale was finalized with 5 subdimensions comprising a total of 18 items. In terms of subdimension breakdown, infertility includes 3 items, GDM-related measurements has 3, insulin resistance before pregnancy has 5, pregnancy insulin resistance has 4, and healthy lifestyle behaviors has 3. Out of the 18 items on the scale, 15 items assess risky behaviors, while the remaining 3 items measure healthy lifestyle behaviors. The expressions related to healthy lifestyle behaviors are reverse-coded in the data set. The difference between the smallest and largest values in the data set is referred to as the range. When data are organized from smallest to largest, the segment containing 1% of the data is referred to as the 1st percentile, while the segment containing 50% is referred to as the 50th percentile, commonly recognized as the median value. The purpose of determining the 25th, 50th, and 75th percentile values of the GEDRISK scale variables is to establish the standardized criteria that will guide assessments in subsequent applications of the scale. Within the scale, it was assumed that a raw score corresponding to the 25th percentile and below is considered high risk, while a raw score corresponding to the 75th percentile and above is considered low risk. In this context, 30 points and below indicate high risk of GDM, 31–33 points indicate medium risk, 34–36 points indicate low risk, and 37 points and above indicate no risk of GDM. The pregnant women who participated in the study scored 5.7 ± 0.7 in infertility, 6.7 ± 1.4 in GDM-related measurements, 9.1 ± 1.7 in insulin resistance before pregnancy, 6.7 ± 1.8 in pregnancy insulin resistance, and 5.2 ± 1.6 in healthy lifestyle. Overall, it was observed that the participants achieved an average score of 33.5 ± 4.7 points on the GEDRISK scale. The OGTT led to a diagnosis of GDM in 104 of the respondents. The scale was able to identify 51% (53) of those diagnosed with GDM as high risk and 78% (81) as medium to high risk.

## 4. Discussion

Initially, a 53-item question pool was developed, from which a 39-item scale was subsequently constructed and validated through expert input and the results of a pilot study [35]. Content validity was established by converting the results obtained using the Lawshe method into quantitative data and calculating the CVR based on input from 36 experts [34]. The content validity threshold exceeded 0.67, indicating a favorable content validity [35]. This finding indicates that the CVI for each item within the scale is high, sufficient, and suitable for the Turkish context.

Following the confirmation of content validity for the 18-item scale, it was administered to a sample group consisting of 652 individuals, which is approximately 16.7 times the number of items. To ensure the homogeneity of the sample, attention was paid to the diversity of such variables as gender, education level, and working status. The scale was administered to all pregnant women who were accessible and visited 6 public hospitals located in different districts on the Anatolian side of Istanbul. Given that GDM can be influenced by individual and cultural variations, it was deemed advantageous to study a highly diverse sample. The KMO coefficient ranges from 0 to 1. For Bartlett’s test, values equal to or above 0.50 and a significance level of p < 0.05 are considered significant [40,41]. Previous studies in the literature have classified KMO coefficients as follows: 0.90 and above as excellent, 0.80–0.90 as very good, 0.70–0.80 as good, and 0.50–0.60 as mediocre. Values below 0.50 are considered unacceptable [18,42,33]. The KMO coefficient of the GEDRISK scale exceeding 0.70 indicates that the adequacy of the research sample is at a good level for conducting a factor analysis [18,33]. The highly significant (p < 0.001) result of Bartlett’s test indicates a relationship between the variables of the GEDRISK scale, demonstrating its effectiveness in identifying the subdimensions of the preliminary scale [43]. Based on the results of the preliminary assumption tests, the sample size was deemed suitable for the KMO test, the Bartlett’s test for the analysis of the relationship between factors, and the antiimage correlation test for the factor analysis of the items [35].

An EFA was conducted to determine the construct validity of the scale. Factor determination methods including a principal components analysis and the varimax method accounted for 57.48% of the variance. A variance value falling within the range of 0.50–0.70, as indicated in the literature, is considered adequate [33,35]. Consequently, the analysis demonstrated that the structure accounts for over half of the variance, underscoring the strength of the factor structure.

Previous studies in the literature have suggested that there should be a minimum of 3 items per factor. Recommendations further indicate that factors with fewer than 3 items should not be classified as factors [44–47]. The 5-factor structure of the GEDRISK scale adheres to these rules, with a minimum of 3 items present in each subdimension.

A further EFA was employed to establish the theoretical structure of the GEDRISK scale, followed by CFA for analytical testing of the obtained model. Accordingly, χ^2^/df showed an acceptable fit [38,46,48], GFI showed a good fit [38,43], AGFI showed a good fit [16,38], RMR showed a good fit [48], NFI showed an acceptable fit [49], CFI showed an acceptable fit [49], RMSEA showed a good fit [48], IFI showed a good fit [48], and RFI showed a good fit [48]. These findings suggest a high level of model-data consistency, which is considered acceptable.

After the factor analysis, a reliability analysis of the 18 items in the scale was conducted, which revealed a Cronbach’s alpha value of 0.756. Furthermore, the presence of items that could enhance reliability was examined by removing items from the scale. No item was identified for removal from the scale, and the overall structure of the scale remained unchanged. Previous studies in the literature have indicated that within the range of Cronbach’s alpha coefficient from 0.00 to 1.00, values between 0.40 and 0.59 indicate low reliability, values between 0.60 and 0.79 indicate moderate reliability, and values between 0.80 and 1.00 represent high reliability [18,35,50]. The Cronbach’s alpha values in the present study were higher than 0.60, indicating that the scale is moderately reliable. In assessing the reliability of all the applied score-based scales, the Spearman–Brown and Guttman values, similar to the alpha coefficient, were examined using a split-half method analysis of the test. Values of 1 or very close to 1 are indicative of a perfect fit [33,51]. The results indicate that the items in the scale were closely related and that the scale was homogeneous and exhibited high internal consistency. There was no statistically significant difference between the total scores of the 41 pregnant women who completed the scale twice, with a two-week interval, in an assessment of temporal consistency using the test–retest method [20,35]. Considering the test–retest results of the GEDRISK scale, the reliability of the scale was established as high and deemed appropriate for conducting sensitive measurements over time. Previous studies in the literature suggest that an item–total score correlation coefficient of 0.30 or higher is desirable [17,18]. The present study found that participants comprehended the statements accurately and provided neutral responses, indicating a high level of item discrimination within the scale. A significant difference was noted in the total scores between the groups that received the GEDRISK scale and were divided into the lower and upper quartiles of 27% (p = 0.000). Following the analysis, the aim is to achieve a statistically significant difference between the groups (p < 0.05) [35]. Hence, the items within the scale were considered capable of differentiating between pregnant women at risk of GDM and those without risk [35,52].

The outcome of this study is the development of a measurement tool that can be considered appropriate for the sociocultural structure of Turkey. In this regard, the study fills a previous knowledge gap, both in national and international literature, by introducing a valid and reliable method for assessing the risk of GDM. The study adhered to all the principles of scale development, and one of its key strengths lies in its alignment with Turkish society and language. Another strength of the study is that the sample size was approximately 17 times larger than the recommended size. Therefore, GEDRISK scale scores can be considered appropriate for use by healthcare professionals in Türkiye, including primary healthcare providers, gynecologists and obstetricians, nurses, and midwives working in various healthcare facilities, to predict the risk of GDM in pregnant women. The GEDRISK scale provides an option for pregnant women who choose not to undergo the OGTT procedure or face difficulties in accessing healthcare facilities that offer OGTT. This scale empowers pregnant women to self-assess and determine their own GDM risk levels. It is believed that the scale will encourage high-risk pregnant women to consider undergoing OGTT. Furthermore, the scale can be adapted to various languages and potentially be utilized internationally, making a valuable contribution to the existing literature.

The primary limitation of the present study is that it exclusively involved pregnant women who presented to a state hospital and 5 training and research hospitals in İstanbul, selected through a random sampling method.

## 5. Conclusion

The GEDRISK scale, comprising 18 items distributed across five subdimensions, has demonstrated its validity and reliability. The GEDRISK scale offers an option for pregnant women who prefer not to undergo OGTT or face challenges accessing healthcare facilities offering OGTT, enabling them to self-assess and determine their own GDM risk levels. It is anticipated that pregnant women using the GEDRISK scale to assess their diabetes risk may be more readily encouraged to undergo OGTT if they identify themselves as high-risk individuals.

## Figures and Tables

**Table 1:** Distribution of sociodemographic data of pregnant participants.

Sociodemographic characteristics	Mean	Standard deviation
Age (years)	29.1	5.3
Marriage period (years)	5.9	5.0
	Count (n)	Percent (%)
**Educational status**		
Not literate	12	1.8
Literate	11	1.7
Primary school	184	28.2
High school	235	36.0
University and above	210	32.2
**Job**		
Housewife	508	77.9
Laborer	47	7.2
Civil servant	38	5.8
Healthcare professional	29	4.4
Self-employed	30	4.6
**Health insurance**		
Available	585	89.7
None	67	10.3
**Income rate**		
Income less than expenses	230	35.3
Income equivalent to expense	368	56.4
Income more than expenses	54	8.3

**Table 2:** Mean values of factor items in the GEDRISK scale and factor load distribution.

Factor name	Variance (%)	Substances	Min.	Max.	Mean	SD	Factor load values
Infertility	13.89	2–4	1.00	2.00	1.92	0.24	0.752–0.926
GDM-related measurements	11.74	10–12	1.00	3.00	2.24	0.48	0.571–0.882
Insulin resistance before pregnancy	11.68	14–18	1.00	3.00	1.82	0.35	0.559–0.646
Pregnancy insulin resistance	10.59	35–38	1.00	3.00	1.67	0.45	0.614–0.705
Healthy lifestyle behaviors	9.56	32–34	1.00	3.00	1.74	0.56	0.691–0.742

Min = minimum, Max = maximum, SD = standard deviation.

**Table 3:** Confirmatory factor analysis index values of the GEDRISK scale.

Compliance index*	Ideal compliance	Acceptable compliance	Research findings	Interpretation
RMSEA	0 < RMSEA < 0.05	0.05 < RMSEA < 0.10	0.04	Good fit
NFI	0.95 ≤ NFI ≤ 1	0.90 ≤ NFI ≤ 0.95	0.92	Acceptable fit
CFI	0.97 ≤ CFI ≤ 1	0.95 ≤ CFI ≤ 0.97	0.95	Acceptable fit
IFI	0.95 ≤ IFI ≤ 1	0.90 ≤ IFI ≤ 0.95	0.95	Good fit
RFI	0.90 ≤ RFI ≤ 1	0.85 ≤ RFI ≤ 0.90	0.90	Good fit
RMR/SRMR	0 ≤ SRMR ≤ 0.05	0.05 ≤ SRMR ≤ 0.10	0.01	Good fit
GFI	0.95 ≤ GFI ≤ 1	0.90 ≤ GFI ≤ 0.95	0.95	Good fit
AGFI	0.90 ≤ AGFI ≤ 1	0.85 ≤ AGFI ≤ 0.90	0.93	Good fit
χ^2^/df	0 ≤ χ^2^/df ≤ 2	2 ≤ χ^2^/df ≤ 5	2.28	Acceptable fit
p value	0.05 ≤ p ≤ 1.00	0.01 ≤ p ≤ 0.05	p < 0.001	

* RMSEA = root mean square error of approximate, NFI = normed fit index, CFI = comparative fit index, IFI = incremental fit index, RFI = relative fit index, RMR/SRMR = root mean square residual/standardized RMR, GFI = goodness of fit index, AGFI = adjusted goodness of fit index, and χ^2^/df = adjusted chi-square statistic.

**Table 4:** Cronbach’s alpha and item–total statistics.

GEDRISK items	*r	**p	Scale mean if item deleted	Scale variance if item deleted	Corrected item–total correlation	Cronbach’s alpha if item deleted	Subdimensions and Cronbach’s alpha
1	0.46	0.000	31.66	21.112	0.416	0.747	Infertility 0.90
2	0.46	0.000	31.65	21.141	0.416	0.748	
3	0.54	0.000	31.63	21.098	0.508	0.746	
4	0.47	0.000	31.24	20.061	0.313	0.748	GDM-related measurements 0.62
5	0.43	0.000	31.18	20.033	0.373	0.743	
6	0.37	0.000	31.56	20.597	0.259	0.752	
7	0.46	0.000	31.69	20.422	0.384	0.744	Insulin resistance before pregnancy 0.74
8	0.38	0.000	31.93	20.494	0.278	0.751	
9	0.43	0.000	31.77	20.088	0.312	0.748	
10	0.53	0.000	31.67	19.999	0.450	0.739	
11	0.47	0.000	31.68	19.866	0.363	0.744	
12	0.42	0.000	31.90	19.828	0.283	0.753	Healthy lifestyle behaviors 0.60
13	0.46	0.000	31.83	19.388	0.315	0.750	
14	0.47	0.000	31.74	19.542	0.345	0.746	
15	0.34	0.000	32.11	20.649	0.216	0.756	Pregnancy insulin resistance 0.62
16	0.49	0.000	31.85	19.582	0.378	0.743	
17	0.53	0.000	31.78	19.156	0.415	0.739	
18	0.52	0.000	31.84	19.492	0.414	0.739	

* r = Pearson correlation analysis,

** = p < 0.05.
